# Relative effects of breastfeeding intention and practice on maternal responsiveness

**DOI:** 10.1002/imhj.21832

**Published:** 2019-09-25

**Authors:** Catherine L. Jones, Iryna Culpin, Jonathan Evans, Rebecca M. Pearson

**Affiliations:** ^1^ Department of Psychology School of Life Sciences University of Exeter Exeter United Kingdom; ^2^ Centre for Academic Mental Health Population Health Sciences Bristol Medical School University of Bristol Bristol United Kingdom

**Keywords:** Avon Longitudinal Study of Parents and Children, breastfeeding intention, breastfeeding practice, maternal responsiveness, parent–child interactions, Estudio Longitudinal Avon de Padres y Niños, intención de amamantar, práctica de amamantar, interacciones padres‐niño, sensibilidad materna, Etude Longitudinale de Parents et d'Enfants de l'Avon, intention d'allaitement, pratique d'allaitement, interactions parent‐enfant, réaction maternelle, Avon‐Längsschnittstudie von Eltern und Kindern, Stillabsicht, Stillpraxis, Eltern‐Kind‐Interaktion, mütterliche Responsivität, Avon 両親 • ・子ども調査縦断研究, 母乳育児の意向, 母乳育児の実施, 親−子交流, 母親の応答性, Avon父母和兒童縱向研究, 母乳喂養意圖, 母乳喂養習慣, 親子互動, 母體反應, دراسة افون الطولية للوالدين والأطفال, نية الرضاعة الطبيعية, ممارسه الرضاعة الطبيعية, تفاعل الوالدين والطفل, استجابه الأمهات

## Abstract

Our objective was to examine the differential effects of antenatal breastfeeding intention (BI) and breastfeeding practice (BP) on maternal postnatal responsiveness. We conducted a secondary analysis of longitudinal data from a subsample of 962 mother–infant dyads from a U.K.‐based birth cohort study the Avon Longitudinal Study of Parents and Children. Exposures were BI and BPs measured at 32 weeks of gestation and 18 months’ postpartum. The outcome was maternal responsiveness assessed at 12 months’ postpartum. We used logistic regression analyses unadjusted and adjusted for confounders. Intention to breastfeed was associated with increased odds of postnatal maternal responsiveness independent of BP, adjusted odds ratio (OR) = 2.34, 95% CI [1.42, 3.86]. There was no evidence that BP was an independent predictor of maternal responsiveness, OR = 0.93, 95% CI [0.55, 1.57]. Life‐course epidemiology analyses demonstrated that maternal responsiveness is most positive when both BI and BP are present. This is the first population‐based study to provide evidence that BI during pregnancy is more strongly associated with maternal postnatal responsiveness than is BP. Further research is needed to understand the determinants of BI in pregnancy and its relationships with maternal responsiveness.

## RESUMEN

**Propósito**: nuestro objetivo fue examinar los efectos diferenciales de la intención antenatal de amamantar y la práctica de amamantar sobre la sensibilidad materna posnatal.

**Métodos**: llevamos a cabo un análisis secundario de información longitudinal de un subgrupo muestra de 962 díadas madre‐infante que eran parte de un estudio británico de cohorte de nacimiento, el Estudio Longitudinal Avon de Padres y Niños. Los aspectos de exposición fueron la intención de amamantar y las prácticas de amamantar según fueron medidas a las 32 semanas de gestación y 18 meses posparto. El resultado fue la sensibilidad materna evaluada a los 12 meses posparto. Usamos análisis de regresión logística sin ajustar y ajustados para factores de confusión.

**Resultados**: se asoció la intención de amamantar con mayores probabilidades de sensibilidad materna posparto independiente de la práctica de amamantar (ajustada proporción de probabilidades (OR) 2.34, 95% CI 1.42, 3.86). No hubo evidencia de que la práctica de amamantar fuera un independiente factor de predicción de la sensibilidad materna (OR 0.93, 95% CI 0.55, 1.57). Los análisis epidemiológicos de curso vital demostraron que la más positiva sensibilidad materna se da cuando ambas, la intención de amamantar y la práctica de amamantar, están presentes.

**Conclusiones**: este es el primer estudio con base en la población que aporta evidencia de que la intención de amamantar durante el embarazo está más fuertemente asociada con la sensibilidad materna posparto que la práctica de amamantar. Mayor investigación es necesaria para comprender los factores determinantes de la intención de amamantar durante el embarazo y sus relaciones con la sensibilidad materna.

## RÉSUMÉ

**But** Notre objectif était d'examiner les effets différentiels de l'intention d'allaitement au sein avant la naissance et la pratique d'allaitement au sein sur la réaction maternelle postnatale.

**Méthodes** Nous avons procédé à une analyse secondaire de données longitudinales à partir d'un sous‐échantillon de 962 dyades mère‐nourrisson d'une étude de cohorte de naissance britannique, l'Etude Longitudinale de Parents et d'Enfants de l'Avon. Les risques étaient l'intention d'allaitement au sein et les pratiques d'allaitement mesurées à 32 mois de grossesse et à 18 mois après la naissance. Le résultat était la réaction maternelle évaluée à 12 mois postpartum. Nous avons utilisé des analyses de régression logistique non ajustées et ajustées pour les facteurs de confusion.

**Résultats** L'intention d'allaiter au sein était liée à des chances accrues de réaction maternelle postnatale sans lien à la pratique d'allaitement (rapport de cote ajusté (RCaj) 2,34, 95% CI 1,42, 3,86). On n'a trouvé aucune preuve que la pratique d'allaitement était un facteur de prédiction indépendant de la réaction maternelle (RCaj 0,93, 95% CI 0,55, 1,57). Les analyses d’épidémiologie du parcours ont démontré que la réaction maternelle est plus positive lorsque l'intention d'allaitement et la pratique d'allaitement sont présentes.

**Conclusions** Voici la première étude sur une population qui présente des preuves que l'intention d'allaitement durant la grossesse est plus fortement liée à la réaction postnatale maternelle que la pratique d'allaitement. Des recherches plus approfondies sont nécessaires afin de comprendre les déterminants de l'intention de l'allaitement durant la grossesse et sa relation à la réaction maternelle.

## ZUSAMMENFASSUNG

**Zielsetzung** Unser Ziel war es, die unterschiedlichen Auswirkungen von vorgeburtlicher Stillabsicht und Stillpraxis auf die postnatale Responsivitätt von Müttern zu untersuchen.

*Methoden* Wir führten eine Sekundäranalyse der Längsschnittdaten einer Teilstichprobe, bestehend aus 962 Mutter‐Kind‐Dyaden, aus einer britischen Geburtskohortenstudie durch, der Avon‐Längsschnittstudie von Eltern und Kindern. Die unabhängigen Variablen waren die Stillabsicht und die Stillpraxis, gemessen in der 32. Schwangerschaftswoche und 18 Monate nach der Geburt. Die abhängige Variable war die Responsivität der Mütter, die 12 Monate nach der Geburt beurteilt wurde. Wir haben sowohl nicht angepasste logistische Regressionsanalysen verwendet als auch solche, die für Störfaktoren angepasst wurden.

**Ergebnisse** Die Absicht zu stillen war mit einer erhöhten mütterlichen Responsivität assoziiert, unabhängig von der postnatalen Stillpraxis (angepasstes odds ratio (OR) 2.34, 95% CI 1.42, 3.86). Es gab keine Hinweise darauf, dass die Stillpraxis ein unabhängiger Prädiktor für mütterlichen Responsivität war (OR 0.93, 95% CI 0.55, 1.57). Life‐course epidemiologische Analysen haben gezeigt, dass die Responsivität der Mütter am positivsten ist, wenn sowohl die Absicht zum Stillen als auch die Stillpraxis vorliegen.

**Fazit** Dies ist die erste bevölkerungsbasierte Studie, die nachweisen konnte, dass die Stillabsicht während der Schwangerschaft stärker mit der postnatalen Responsivität der Mutter zusammenhängt als die Stillpraxis. Weitere Studien sind erforderlich, um die Determinanten von Stillabsicht in der Schwangerschaft und ihre Beziehung zur Responsivität der Mutter zu untersuchen.

## 抄録

**母親の応答性に対する母乳育児の意向と実施の相対効果**

**目的** 私たちの目的は、出生後の母親の応答性に対する出生前の母乳育児の意向と母乳育児実施の効果差 について調査することであった。

**方法** 私たちは、英国を拠点とした出生コホート研究「Avon 両親・子ども調査縦断研究」から 962 組の母子をサブサンプリングとし、縦断的データの二次分析を実施した。曝露因子は、妊娠 32 週 と産後 18 週に測定した母乳育児の意向と母乳育児の実施であった。 アウトカムは、産後 12 ヶ月に評価された母親の応答性であった。交絡因子で補正されていないロジスティック回帰分析と補正されたものとを使用した。

**結果** 母乳育児への意向は、母乳育児の実施とは独立して、産後の母親の応答性と高い確率で関連していた(補正されたオッズ比 (OR) 2.34, 95% CI 1.42, 3.86)。母乳育児の実施が、母親の応答性の独立した予測因子だという証拠はなかった (OR 0.93, 95% CI 0.55, 1.57)。 ライフコース疫学分析は、母乳育児への意向と実施が両方ある時に、母親の応答性がおおよそポジティブになると示していた。

**結論** これは、妊娠中の母乳育児の意向が、母乳育児の実施よりも、より強く母親の産後の応答性と関係していることを証明した最初の集団ベース研究である。妊娠中の母乳育児への意向の決定要因と母親の応答性との関係性を理解するためには更なる研究が必要である。

## 摘要

母乳喂養方式和實踐對母親反應能力的相對影響

**目的** 我們的目的是檢查產前母乳喂養意願和母乳喂養習慣對母親產後反應的不同影響。

**方法** 我們對來自英國出生隊列的 962 個母嬰二元組子樣本的縱向樣本進行了縱向數據的二手分析, 該子群組來自Avon 縱向父母與子女研究。暴露是在懷孕 32 周和產後 18 個月測量的母乳喂養意願和母乳喂養習慣。結果是在產後 12 個月評估母體反應。我們使用了未經校正和針對混雜因素進行校正的邏輯回歸分析。

**結果** 獨立於母乳喂養的習慣, 母乳喂養的意願與產後母親反應的機率增加相關 (校正比值比) OR (為2.34, 95％CI 1.42, 3.86) 。沒有證據表明母乳喂養是孕婦反應能力的獨立預測因子 (OR 0.93, 95％CI 0.55, 1.57)。生命週期流行病學分析表明, 當同時存在母乳喂養意圖和母乳喂養習慣時, 母親反應最為積極。

**結論** 這是第一項以人群為基礎的研究, 提供的證據表明, 懷孕期間的母乳喂養意願與母體產後反應能力比母乳喂養實踐更緊密相關。需要進一步的研究來了解妊娠中母乳喂養意願的決定因素及其與母親反應能力的關係。

## ملخص

**الآثار النسبية لنية الرضاعة الطبيعية وممارستها علي الاستجابة الأمومية **

**الغرض **: الهدف من الدراسة هو دراسة الآثار التفاضلية لنية الرضاعة الطبيعية قبل الولادة وممارسة الرضاعة الطبيعية علي استجابه الأمهات بعد الولادة.

**منهج البحث ** : أجرينا تحليلا ثانويا للبيانات الطولية من عينه فرعيه من 962 من ثنائيات الأمهات والرضع من واقع دراسة ولادة في المملكة المتحدة (دراسة أفون الطولية للآباء والأطفال). وكانت نية الرضاعة الطبيعية وممارسات الرضاعة الطبيعية تقاس عند 32 أسبوعا من الحمل و 18 شهرا بعد الولادة. وكان المتغير التابع هو استجابة الأمهات التي تم تقييمها بعد 12 شهرا بعد الولادة. استخدمنا تحليلات الانحدار اللوجستية غير المعدلة والمعدلة وفقا للمتغيرات المناقضة.

**النتائج ** : ارتبطت نية الرضاعة الطبيعية بزيادة احتمالات استجابه الأمهات بعد الولادة بشكل مستقل عن ممارسه الرضاعة (نسبه الاحتمالية المعدلة (OR) 2.34 ، 95% CI 1.42 ، 3.86). ولم يكن هناك دليل علي ان ممارسه الرضاعة الطبيعية كانت مؤشرا مستقلا لاستجابه الأمهات (معدل الاحتمالية 0.93 ، 95 ٪ CI 0.55 ، 1.57). وأظهرت التحاليل الحيوية ان الاستجابة الأمومية تكون في أكثر حالاتها الإيجابية عندما تكون النية للرضاعة الطبيعية وممارسه الرضاعة موجودة.

**الاستنتاجات **: وهذه هي أول دراسة تستند إلى السكان لتقديم الادله علي ان نية الرضاعة الطبيعية اثناء الحمل ترتبط ارتباطا اقوي باستجابة الأمهات بعد الولادة من ممارسه الرضاعة الطبيعية. وهناك حاجه إلى مزيد من البحوث لفهم العوامل المحددة لنية الرضاعة الطبيعية في الحمل وعلاقاتها مع استجابه الأمهات.

## 1 INTRODUCTION

Sensitivity is one of the key constructs of attachment theory (Bowlby, 1969; Shin, Park, Ryu, & Seomun, 2008). Ainsworth, Blehar, Waters, & Wall (1987) defined maternal sensitivity as a mother's ability to perceive and interpret accurately her infant's signals and communications and then respond appropriately. Empirical research has identified sensitivity as an important, but not exclusive, predictor of secure infant attachment (Bigelow et al., 2010; De Wolff & van IJzendoorn, 1997). Similarly, the causal role of maternal responsiveness on infant brain development has been directly demonstrated in nonhuman animal research (Eshel, Daelmans, de Mello, & Martines, 2006). Observations of mothers showing positive maternal responsiveness toward their infants provide a core index of maternal sensitivity. Evidence has suggested that maternal responsiveness is associated with the later emotional, cognitive, and physical development of the infant (De Wolff & van IJzendoorn, 1997; Murray, Fiori‐Cowley, Hooper, & Cooper, 1996). Thus, insights into the factors associated with maternal responsiveness may inform programs aimed at promoting healthy child development with breastfeeding as a potentially important consideration.

The benefits of breastfeeding for the child are well‐documented (Ivarsson, Hernell, Stenlund, & Persson, 2002; Kramer et al., 2001). The World Health Organization has recommended 6 months of exclusive breastfeeding for the infant to benefit from the positive effects of breastfeeding. However, the effect of breastfeeding on maternal responsiveness remains undetermined. Existing research has mostly focused on the effect of breastfeeding on infant development and attachment rather than on maternal responsiveness (Heikkila, Sacker, Kelly, Renfrew, & Quigley, 2011; Kramer et al., 2008). Although positive effects of breastfeeding on maternal responsiveness are often advocated, empirical evidence is lacking (Jansen, de Weerth, & Riksen‐Walraven, 2008).

Theoretically, breastfeeding may enhance maternal responsiveness via a number of mechanisms. For instance, suckling stimulates the endocrine system to release oxytocin and prolactin, which have been linked to indices of maternal sensitivity (e.g., licking and grooming) in animal research (Kendrick, 2000; Roberts, Jenkins, Lawler, Wegner, & Newman, 2001). In addition to biological effects, behavioral aspects of breastfeeding may also enhance maternal responsiveness and infant attachment through increased sensory interactions and touch (Widstrom et al., 1990). Breastfeeding has also been shown to positively affect maternal mood, which may in turn promote maternal sensitivity (Mezzacappa, Kelsey, & Katkin, 2005). Contextual factors such as family pressure, social and cultural expectations, and health professionals’ support may also influence mother's breastfeeding practices (BPs). In addition, it may be that mothers who intend to breastfeed are by nature more responsive; however, few studies have examined the characteristics of women who choose to breastfeed in this context.

Britton, Britton, and Gronwaldt (2006) examined the relationship between antenatal breastfeeding intention (BI), BP, maternal sensitivity, and infant attachment. Maternal sensitivity was measured via observational ratings of the quality of mother–infant interactions at 6 months. Both BI and BP were found to positively correlate with maternal sensitivity; however, the independent effect of these factors was not tested (Britton et al., 2006). Pearson, Lightman, and Evans (2011) demonstrated that breastfeeding is associated with enhanced maternal sensitivity to infant distress, as compared to those mothers who bottle‐fed, and that this difference emerges only after birth once feeding has commenced. Conversely, Drake et al. (2007) examined potential predictors of maternal sensitivity and found that breastfeeding was not associated with maternal sensitivity as rated by subjective reports, however, self‐esteem, satisfaction with life and parity emerged as significant factors. In a recent neuroimaging study, Kim et al. (2011) demonstrated links between breastfeeding and greater maternal responses to infant cues in brain regions implicated in maternal–infant bonding and empathy during the early postnatal period.

However, there remains a lack of longitudinal research on breastfeeding and maternal responsiveness using large population‐based samples while accounting for possible confounding factors, particularly those that may influence mother's decision to breastfeed in pregnancy. In addition, it may be that breastfeeding, per se, enhances maternal responsiveness; alternatively, mothers who practice breastfeeding may have higher preexisting levels of responsiveness versus mothers without BI. Disentangling BI and BP requires large sample sizes due to high correlation between these factors, and a need to include rarer categories (i.e., women without intention to breastfeed who practice breastfeeding after birth).

Our objective was to examine the differential effects of prenatal BI and BP on maternal postnatal responsiveness. We used prospectively collected data from the Avon Longitudinal Study of Parents and Children (ALSPAC), a large birth cohort in Bristol, to address some of the existing gaps in the literature. The relatively large sample size enabled us to investigate subgroups of women according to their BI and BP over time (i.e., women with/without BI who did/did not practice breastfeeding) using a life‐course model‐building approach (Mishra et al., 2009). Our research questions were:

**RQ1**: Do women with intention to breastfeed who did breastfeed show a greater proportion of positive (vs. neutral) maternal responses, as compared to the other groups?
**RQ2**: Is BP a critical factor in predicting maternal responsiveness (i.e., women without intention to breastfeed who did breastfeed show a greater proportion of positive [vs. neutral] maternal responses, as compared to women with intention to breastfeed who did not breastfeed)?

## 2 MATERIALS AND METHODS

### 2.1 Data source

The sample comprised participants from the Avon Longitudinal Study of Parents and Children (ALSPAC). During Phase I enrolment, 14,541 pregnant mothers residing in Avon, United Kingdom with expected dates of delivery from April 1, 1991 to December 31, 1992 were recruited. These pregnancies resulted in 14,062 live births and 13,988 children who were alive at 1 year of age. The current study comprises a 10% subsample of the ALSPAC children, known as the “Children in Focus” (CiF) group, who attended clinics at the University of Bristol. The CiF group members were chosen at random from the final 6 months of ALSPAC births. In total, 1,213 parent–infant pairs attended a 12‐month CiF assessment, and 1,144 participated in videotaped mother–infant interactions. The representativeness of this sample compared with those who did not attend the CiF assessment is shown in Table 1. Detailed information about ALSPAC is available at http://www.bristol.ac.uk/alspac/, including a searchable data dictionary (http://www.bris.ac.uk/alspac/researchers/data-access/data-dictionary/) (for further details on the cohort profile, representativeness, and phases of recruitment, see Boyd et al., 2013; Fraser et al., 2013; and Golding, Pembrey, & Jones, 2001).

**Table 1:** Sample characteristics (ALSPAC: Avon Longitudinal Study of Parents and Children)

Parental and socioeconomic characteristics	Mothers who did not attend the research clinic but had a live singleton at 12 months (*n* = 12,415)	Mothers who attended the research clinic, but had missing data (*n* = 347)	Complete case sample^a^ (*n* = 894)	*P*‐value	Breastfeeding: intention = yes; practice = yes (*n* = 441)	Breastfeeding: intention = no; practice = yes (*n* = 76)	Breastfeeding: intention = yes; practice = no (*n* = 182)	Breastfeeding: intention = no; practice = no (*n* = 263)	*P*‐value
Maternal age at delivery, *M*	28	28	29	<.001	30	29	28	27	<.001
Maternal educational attainment									
University degree	14%	13%	16%	<.001	24%	16%	9%	3%	<.001
O‐level/A‐level	61%	64%	65%	<.001	63%	63%	65%	61%	<.001
Minimal/no education	16%	12%	12%	<.001	13%	21%	24%	36%	<.001
Parity									
Primiparous	44%	46%	47%	<.001	43%	53%	62%	37%	<.001
Pregnancy intended									
Yes	69%	67%	77%	<.001	74%	70%	72%	68%	<.001

*Note*.

a Complete case sample: Mother–infant pairs with complete data on exposures, outcome, and confounders.

### 2.2 Materials

#### 2.2.1 Outcome variable: Maternal responsiveness

Maternal responsiveness was measured at the 12‐month CiF assessment using the Thorpe Interaction Measure (TIM; Thorpe, Rutter, & Greenwood, 2003). Mothers were asked to share a picture book with the child as they would normally do at home for approximately 5 min. The sensitivity of maternal nonverbal responses to her infant was rated by an independent researcher during the observation in accordance with the protocol. The interrater reliability of κ ≥ 0.6 across four raters had to be established for all behavioral codes within the coding system (Thorpe et al., 2003). The study focused on nonverbal rather than on verbal maternal responses, given that these behaviors may reflect instinctive and automatic responses less likely to be affected by social desirability bias. TIM nonverbal behavioral codes compare well to those used to code maternal sensitivity in other validated maternal‐sensitivity scales (Page, Wilhelm, Gamble, & Card, 2010). Furthermore, there was little variance in maternal verbal responses, with the majority of mothers (80%) showing positive responses.

Previous research has considered maternal verbal responses as separate from the responses used to categorize maternal sensitivity. Using ALSPAC data, we found that positive maternal responses were independently associated with a 0.3 *SD* increase in an experimenter‐assessed infant development score at 18 months after controlling for maternal and infant characteristics (Pearson et al., 2012), thus supporting the inter‐rater reliability and predictive validity of maternal nonverbal responsiveness on infant development.

Maternal nonverbal responses were categorized as *positive* (e.g., stroking, kissing, caressing, positive eye contact, smiling), *neutral* (e.g., no clear example of either negative or positive behavior), or *negative* (e.g., gaze aversion, pushing, distracting, nonresponse to positive initiation). Maternal negative behaviors were rare, and it was deemed inappropriate to combine negative and neutral behaviors given that these responses may be qualitatively different (Thorpe et al., 2003). Thus, we did not include negative responses in the final analyses. Scores were coded as 1 (*positive*) and 0 (*neutral*).

#### 2.2.2 Exposure variables: BI and BP

Information on how the mother intended to feed her baby in the postnatal period was collected at 32 weeks of gestation via mother‐reported questionnaires. The response categories included: “breast,” “bottle,” “breast and bottle,” or “uncertain.” For the initial main effects analysis, we created three BI groups, encompassing “yes” (corresponding to the “breast” category), “maybe” (generated by collapsing the “breast and bottle” and “uncertain” categories), and “no” (corresponding to the “bottle” category). Maternal BP was assessed via mother‐reported questionnaires on the feeding methods at 18 months postnatally (including breastfeeding duration) to capture the full extent of breastfeeding duration across the postnatal period. Thus, mothers who supplemented breastfeeding with formula and later solid food (after 6 months) would still have been classified as breastfeeding. The response categories included: “never,” “less than 3 months,” “3–5 months,” or “6 months+.” Evidence has suggested that that maternal recall is a valid and reliable estimate of breastfeeding initiation and duration, especially when breastfeeding is recalled after a short period of time (Li, Scanlon, & Serdula, 2005).

#### 2.2.3 Confounders

Parental and socioeconomic characteristics identified in previous studies as being associated with breastfeeding and maternal responsiveness were collected prospectively from maternal questionnaires during the antenatal and early postnatal periods. These included maternal age at delivery (in years), highest maternal educational attainment (minimal education or none, compulsory secondary level [up to age 16 years], noncompulsory secondary level [up to age 18 years] vs. university level education), parity (primiparous vs. multiparous), and whether the pregnancy was intentional (yes or no). Analyses were also adjusted for maternal antenatal depression score assessed using the Edinburgh Postnatal Depression Scale (Cox, Holden, & Sagovsky, 1987) at 18 weeks of gestation.

### 2.3 Statistical analyses

#### 2.3.1 Main effects

All analyses were conducted using Stata Version 13 (StataCorp, College Station, TX). First, we examined the main effects and interaction of BI and BP on maternal responsiveness in the complete case sample (*n* = 894) using logistic regression.

#### 2.3.2 Nested models

**Figure 1:**
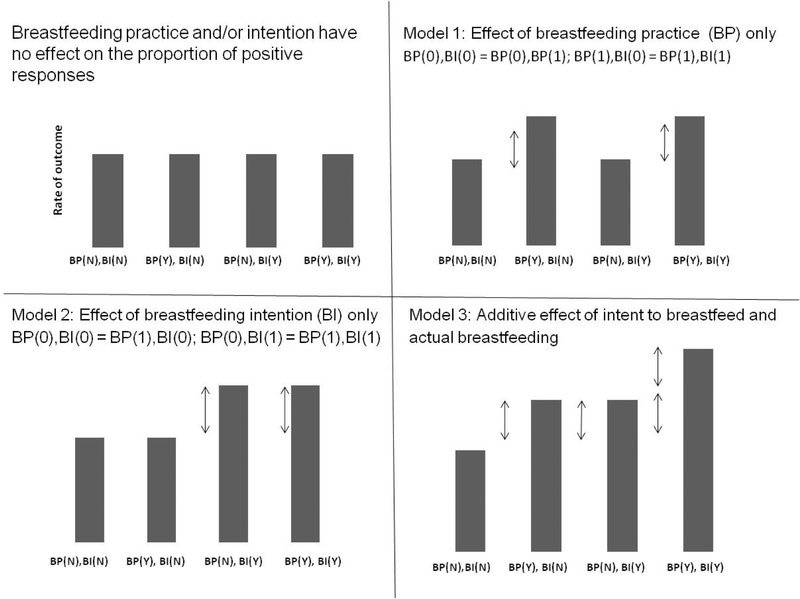
Representation of the four models for comparison using the nested models approach

Second, we explored the relative effects of BI and BP on maternal responsiveness using a life‐course epidemiology approach (Mishra et al., 2009). We performed nested model analyses whereby a fully saturated regression model, which explores all possible patterns of BI and BP, is compared with three nested models using Likelihood‐ratio tests (Figure 1).

##### 2.3.2.1 Model 1: Critical effect of BP

This model tests the hypothesis that only BP will influence maternal responsiveness by restricting the nested model with the following constraints: (a) There is no effect of BI without BP (i.e., this group does not differ from the reference category including those who did not intend to and did not breastfeed: BI:YES/BP:NO = BI:NO/BP:NO); and (b) the effect of practicing breastfeeding with and without intention will be equal (i.e., BI:NO/BP:YES = BI:YES/BP:YES).

##### 2.3.2.2 Model 2: Critical effect of BI

This model tests the hypothesis that only BI will influence maternal responsiveness by restricting the nested model with the following constraints: (a) There is no effect of BP without intention (i.e., BI:NO/BP:YES = BI:NO/BP:NO and BI:NO/BP:YES = BI:YES/BP:YES).

##### 2.3.2.3 Model 3: Additive effect of both BP and BI

This model tests the hypothesis that both BI and BP contribute to maternal responsiveness by restricting the nested model with the following constraint: The sum of the effects of BI only and BP only equals the effects of intending to and actually breastfeeding (i.e., (BI:NO/BP:YES) + (BI:YES/BP:NO) = BI:YES/BP:YES).

Higher *P*‐values and relatively lower Bayesian information criterion (BIC) estimates indicate the relative goodness of fit of the nested model and, thus, whether the hypotheses indicated by the constraints is supported. To increase statistical power, all women with data on BI, BP, and nonverbal responses were included in the nested model analyses (*n* = 962).

#### 2.3.3 Saturated model

We investigated the impact of all potential patterns of BI and BP on maternal sensitivity by creating a four‐level categorical variable. The four levels were: BI: YES and BP: YES (BI:YES/BP:YES); BI: YES and BP: NO (BI:YES/BP:NO); BI: NO and BP: YES (BI:NO/BP:YES); and BI: NO and BP: NO (BI:NO/BP:NO; reference category; Figure 1).

#### 2.3.4 Missing data

The data set contained missing data which varied across variables. Therefore, each analysis was limited to data that was complete for all exposure, outcome, and confounding variables (referred to as *complete case*).

## 3 RESULTS

### 3.1 Sample demographics

**Figure 2:**
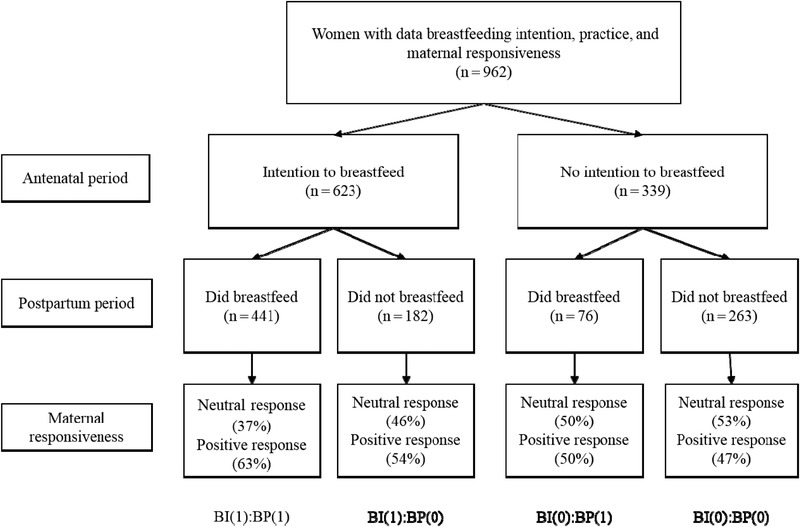
Sample sizes of each category related to breastfeeding intention and practice and respective effects of each of the groups on maternal responsiveness (percentage of mothers demonstrating neutral vs. positive responsiveness)

Our starting sample included 962 women with data on BI, BP, and mother–infant interactions, of which 894 mother–infant pairs had complete data on exposure, outcome, and confounders. Sample sizes of each category related to BI and BP, as well as respective effects of each of the groups on maternal responsiveness, are illustrated in Figure 2. Sample characteristics are provided in Table 1.

### 3.2 Main effects: The association of BI and BP with maternal responsiveness

The logistic regression analyses provided evidence for a main effect of BI and BP on the odds of mothers displaying positive responsiveness (Table 2). Specifically, mothers with BI during pregnancy had higher odds of showing positive maternal responses versus those without BI. There was evidence of a dose–response relationship, with the odds of positive maternal responses increasing as the duration of BP increased.

**Table 2:** Logistic regressions to examine the main effects of breastfeeding intention (vs. no breastfeeding intention) and breastfeeding practice (vs. no breastfeeding practice) on the odds^a^ of mothers displaying positive responsiveness at 12 months’ postpartum

	Unadjusted	Adjusted 1^b^	Adjusted 2^c^
Exposure/Risk group (age of assessment)	OR (95% CI), *P*‐value	OR (95% CI), *P*‐value	OR (95% CI), *P*‐value
Breastfeeding intention (32 weeks of gestation)			
Reference group: No (*n* = 169)	–	–	–
Maybe (*n* = 294)	1.74 (1.19, 2.55), .004	1.86 (1.15, 3.00), .01	1.80 (1.09, 2.92), .02
Yes (*n* = 431)	2.38 (1.66, 3.43), <.001	2.36 (1.44, 3.86), .001	2.34 (1.42, 3.86), .001
Breastfeeding practice (18 months’ postpartum)			
Reference group: Never (*n* = 183)	–	–	–
>3 months (*n* = 229)	1.12 (0.76, 1.66), .56	0.75 (0.47, 1.21), .24	0.73 (0.45, 1.19), .21
3–5 months (*n* = 170)	1.62 (1.06, 2.48), .02	0.99 (0.59–1.67), .98	0.94 (0.55, 1.60), .83
6 months+ (*n* = 312)	1.83 (1.27–2.66), .001	1.03 (0.63–1.70), .90	0.93 (0.55, 1.57), .78

*Note*.

a Odds of showing positive responses: 0 = *neutral*, 1 = *positive*.

b Adjusted for age at delivery, educational attainment, parity, and whether pregnancy had been intended.

c Adjusted for all aforementioned confounders and maternal antenatal depression.

### 3.3 Independent effects of BI and BP on maternal responsiveness

We carried out logistic regression analyses entering both exposure variables (BI and BP) into the same model. There was evidence of an independent effect of BI on maternal responsiveness. In contrast, there was no evidence of an independent effect of BP on maternal responsiveness once BI was accounted for, suggesting that BI explained a larger proportion of the variance in maternal responsiveness. Adjustment for the socioeconomic and maternal confounders made little difference to the parameter estimates (Table 2). No interactions of BI and BP on maternal responsiveness were observed, likelihood ratio χ2(6) = 8.95, *P* = .18.

### 3.4 Comparison of hypotheses

The highest levels of maternal responsiveness were found in those mothers with intention to breastfeed who went on to breastfeed (accumulation effect; Figure 1; Model 3). Thus, we compared the saturated and nested models to examine possible differences between the models (Table 3). Low *P*‐values indicated that there is a difference between the models, suggesting poorer fit of the nested model when compared to the saturated model. Specifically, in Model 1, the critical effect of BP on maternal responsiveness was not as good at predicting the data than was the saturated model, as indicated by poorer model fit. This provides evidence to suggest that that BP alone is not associated with positive maternal responsiveness. Models 2 and 3 did not differ from the saturated model, suggesting similarly good fit to the data. This provides evidence to support the critical effect of BI as well as the additive effect of both BP and BI on maternal responsiveness. Further comparison of BIC model fit indices suggested that maternal responsiveness was most likely to be affected when both BI and BP were present (Model 3; additive effect). This is further illustrated in Figure 2.

**Table 3:** Comparison of nested models representing specific effects of breastfeeding intention and breastfeeding practice with the fully saturated model

Nested models tested	Comparison model	*df*	χ^2^	*P*‐value	BIC
*Model 1*: Critical effect of breastfeeding practice	Saturated model	2	6.2	.046	1.323
*Model 2*: Critical effect of breastfeeding intention	Saturated model	2	4.1	.118	1.321
*Model 3*: Additive effect of both breastfeeding practice and intention	Saturated model	2	0.7	.704	1.317

*Note*. BIC: Bayesian information criterion.

## 4 DISCUSSION

The purpose of this research was to examine whether BP enhances maternal positive responsiveness or whether women who choose to breastfeed are more maternally responsive by nature. We found that BP alone is not sufficient to increase maternal responsiveness and may only be beneficial when there is a prior intention to breastfeed. It may be that women who intend to breastfeed are by nature more responsive and are more likely to choose to breastfeed, which questions the notion of a direct biologically mediated (i.e., oxytocin) causal pathway from BP to maternal responsiveness. This is in line with recent evidence against the notion that oxytocin mediates the relationship between breastfeeding and maternal sensitivity (Tharner et al., 2012).

Although we initially observed an association between breastfeeding duration and maternal responsiveness, this effect diminished once BI was taken into account. In addition, accounting for socioeconomical and parental confounders made little difference to the estimates of the effect of BI on maternal responsiveness. This emphasizes a need for further research to identify other potential determinants of maternal sensitivity, which in turn may influence women's BP. Also note that in the ALSPAC sample, breastfeeding was initiated by 76% of women (Donath, Amir, & the ALSPAC Study Team, 2003), and these rates were higher than breastfeeding rates in the United Kingdom in the year 2000, when the corresponding rates were 69% (Hamlyn, Brooker, Oleinikova, & Wands, 2002). However, despite somewhat higher breastfeeding rates in the ALSPAC sample, the effect of BI on maternal sensitivity should remain the same due to potentially different biological and behavioral mechanisms.

There is growing evidence to suggest that early life experiences of caregiving influence the development of maternal responsiveness (Belsky, Jaffee, Sligo, Woodward, & Silva, 2005; LeCuyer‐Maus, 2000) and that the adult secure attachment style is associated with greater maternal sensitivity (Ward & Carlson, 1995). Thus, it might be that some women may exhibit characteristics, based on their early life experiences, that predispose them to have higher levels of maternal responsiveness and BI in pregnancy. In addition, the development of mother–fetus attachment during pregnancy has been shown to predict maternal sensitivity during the postnatal period (Siddiqui & Hägglof, 2000) and may explain why some mothers change their mind from their original intention to breastfeed during the postnatal period. Furthermore, those mothers who intended to breastfeed, even though they did not breastfeed, may be more likely to engage in some behaviors associated with breastfeeding (e.g., close proximity and physical contact), which may be important in explaining the association between breastfeeding and responsiveness. Thus, breastfeeding may still be associated with responsiveness, albeit indirectly, through sensitive behaviors that women who intended to breastfeed tend to practice.

Note that mothers who decide to bottle‐feed do so most often because of mother‐centered reasons whereas breastfeeding mothers do so for infant‐centered reasons (Arora, McJunkin, Wehrer, & Kuhn, 2000), thus suggesting preexisting differences in maternal attitudinal and intrapersonal characteristics (e.g., age, socioeconomic status, ethnicity, smoking status, and employment) (Van Esterik, 2002). Societal attitudes toward breastfeeding may also moderate the link between breastfeeding and maternal responsiveness. The decision to breastfeed is often influenced by existing cultural perceptions as well as difficulties associated with integrating breastfeeding into employment and daily activities (Van Esterik, 2002). In some societies, breastfeeding is the norm of infant feeding practice and therefore may not necessarily predict the quality of maternal care whereas in other societies it may not be strongly encouraged (Dennis, 2002). Thus, the decisions associated with infant feeding are complex and are likely to differ across social groups and cultures. In addition, other maternal and infant characteristics such as maternal postnatal depression, infant temperament and feeding difficulties may influence both maternal decision to breastfeed and her responsiveness to the infant. However, conceptually and temporarily, these factors are likely to explain (i.e., lie on the causal pathway), rather than confound, the association between decision to breastfeed (despite the initial intention) and maternal responsiveness. The explanatory role of these potential mechanisms merits further research, which was outside the scope of this study.

The strengths of this study include the large sample size, the observed measure of maternal responsiveness, the availability of rich data on several exposures and confounders, and a longitudinal design that enabled us to examine differential effects of BI and BP using a life‐course approach.

The findings need to be interpreted in light of several limitations. First, the rarer group (women without intention to breastfeed who did breastfeed) was relatively small, reducing the statistical power to detect differences. We did not have information (e.g., negative initial breastfeeding experiences) to explain such a pattern. Second, we were unable to provide information on why women with intention to breastfeed went on to bottle‐feed. This could be explained by a number of reasons, including physical difficulties, social pressures, and work commitments. Such information may be important in advancing our understanding regarding the nature of differences between groups of women with different BIs and BPs. These findings should also be interpreted in light of the increased rate of breastfeeding initiation that took place over the past 20 years. However, in the ALSPAC sample, breastfeeding rates were already somewhat higher than the corresponding average national rates (Donath et al., 2003; Hamlyn et al., 2002) back in the early 1990s, suggesting that our findings are relevant even in the context of contemporary BPs. In addition, future research is required to provide further insights into breastfeeding patterns and to examine potential mechanisms that explain the association between BI, BP, and maternal responsivenss.

These findings have important implications for health policy and intervention development. Considering that the beneficial effects of breastfeeding on the mother–infant relationship are often implicitly assumed and advocated, despite limited empirical evidence, it may be reassuring to mothers that the sensitive nature of their interactions may not be dependent on BP. Nevertheless, breastfeeding is crucial for healthy infant development; thus, identifying the characteristics of women without intention to breastfeed in pregnancy may be an important avenue for developing interventions to improve maternal sensitivity during the antenatal period, and to encourage BP.

## CONFLICT OF INTEREST

The authors declare that they have no conflict of interest.
